# Metabolic signatures in sciatic nerve of PMP22 transgenic rats provide insights into the pathogenesis of charcot-marie-tooth disease type 1 A

**DOI:** 10.1038/s41598-025-31633-7

**Published:** 2026-01-08

**Authors:** Alban Muller, Kerri Grove, Isabelle Christen, Julie Kreider, Camille Santos, Shaila Hoque, Michael Bidinosti, Shinji Hatakeyama, Juan Zhang

**Affiliations:** 1 Discovery Sciences, Novartis Biomedical Research, Basel, Switzerland; 2 Global Discovery Chemistry, Novartis Biomedical Research, Emeryville, USA; 3 Forensische Chemie und Toxikologie, Institut für Rechtsmedizin, Universität Basel, Basel, Switzerland; 4 Neuroscience, Novartis Biomedical Research, Basel, Switzerland; 5 Diseases of Aging & Regenerative Medicine, Novartis Biomedical Research, Basel, Switzerland; 6 Global Discovery Chemistry, Novartis Biomedical Research, Basel, Switzerland

**Keywords:** Lipid metabolism, Peripheral myelin protein 22, Charcot–Marie–Tooth type 1A, Targeted metabolomics, Imaging mass spectrometry, Metabolomics, Myelin biology and repair

## Abstract

Charcot–Marie–Tooth Type 1 A (CMT1A) is a hereditary neuropathy caused by a duplication of the peripheral myelin protein 22 (PMP22) gene. Emerging evidence suggests that lipid metabolism plays a central role in CMT1A pathology. This study investigated metabolic profiles in sciatic nerve tissue and plasma of PMP22 transgenic (TG) and wild-type (WT) rats at 2, 4, and 6 months of age. Utilizing targeted metabolomics, more than 600 metabolites covering central metabolic pathways and major lipid classes were analyzed, revealing distinct age-dependent changes in metabolic pathways. Alterations that emerged early and became increasingly pronounced with age were observed in sphingolipids and glycerophospholipids, while changes in other metabolic pathways, such as amino acids, storage lipids, bile acids, and nucleotide metabolism, were age-specific. Notably, in contrast to these age-dependent adaptive changes, three lipid signatures were identified that remained stable from the earliest age examined. These include: (1) an elevated ratio of hydroxylated to non-hydroxylated sphingolipids, (2) a reduced ratio of monounsaturated-containing to saturated fatty acid containing phosphatidylcholines, and (3) a decreased ratio of hexosylceramides to ceramides. Imaging mass spectrometry analyses confirmed disruptions in sphingolipid metabolism. These findings suggest a key regulatory role of PMP22 in lipid metabolism, as demonstrated by the early stabilization of specific lipid signatures compared to other metabolic changes that occurred in an age-dependent and adaptive manner. These observations provide valuable insights into the pathogenic mechanisms underlying CMT1A.

**Supplementary Information:**

The online version contains supplementary material available at 10.1038/s41598-025-31633-7.

## Introduction

Charcot–Marie–Tooth type 1 A (CMT1A) is the most common inherited neuromuscular disorder affecting the peripheral nervous system (PNS). It is primarily characterized by dysmyelination, which leads to muscle weakness and atrophy^1–7^. A duplication of chromosome 17p11.2, resulting in overexpression of the peripheral myelin protein 22 (PMP22) gene has been identified as the genetic cause of CMT1A^8–11^. PMP22 is a transmembrane protein highly expressed in myelinating Schwann cells of the peripheral nervous system^12–14^. It is also a structural component of the myelin sheath and is essential for myelin production in Schwann cells. Additionally, PMP22 plays critical roles in the formation of plasma membrane structure as well as in regulating lipid metabolism and lipid storage^14–19^.

Growing evidence suggests that individuals with CMT1A exhibit metabolic changes, including dysregulated lipid metabolism, oxidative stress, and mitochondrial dysfunction, which may contribute to disease pathogenesis, progression, and severity^18,20–23^. Abnormal levels of specific lipid species, including sphingolipids (SP or SL), glycerophospholipids (GP), cholesterol, and metabolites of glucose metabolism have been reported in individuals with CMT1A^3,5,6,18,21,22,24^. These metabolic alterations may reflect an underlying molecular dysfunction that contributes to nerve damage and muscle weakness associated with CMT1A.

A comprehensive understanding of the metabolic profiles associated with PMP22 overexpression in peripheral nerve tissue and systemic circulation is important for advancing the development of therapies for peripheral neuropathy and identifying potential biomarkers. In this study, we aimed to identify specific metabolic pathways altered in CMT1A by conducting targeted metabolic profiling of sciatic nerve tissue from a PMP22 transgenic rat model^25^. Applying this approach, we sought to gain a better understanding of the mechanisms underlying the disease and insights into its pathophysiology.

## Results

The PMP22 transgenic (TG) rat, a model of Charcot–Marie–Tooth type 1 A disease, was used to characterize the metabolic profile in both female and male rats at 2, 4, and 6 months of age. Both genders exhibited a similar disease phenotype. While gender differences in metabolic profile were observed, the key lipid signatures were comparable between the two gender groups, with male rats showing higher inter-individual variability. As gender-specific differences in the metabolic profiles are not the focus of this study, the analysis and discussions are focused on female rats, while the main findings from male rats are presented in the Supplementary Material.

**Fig. 1:**
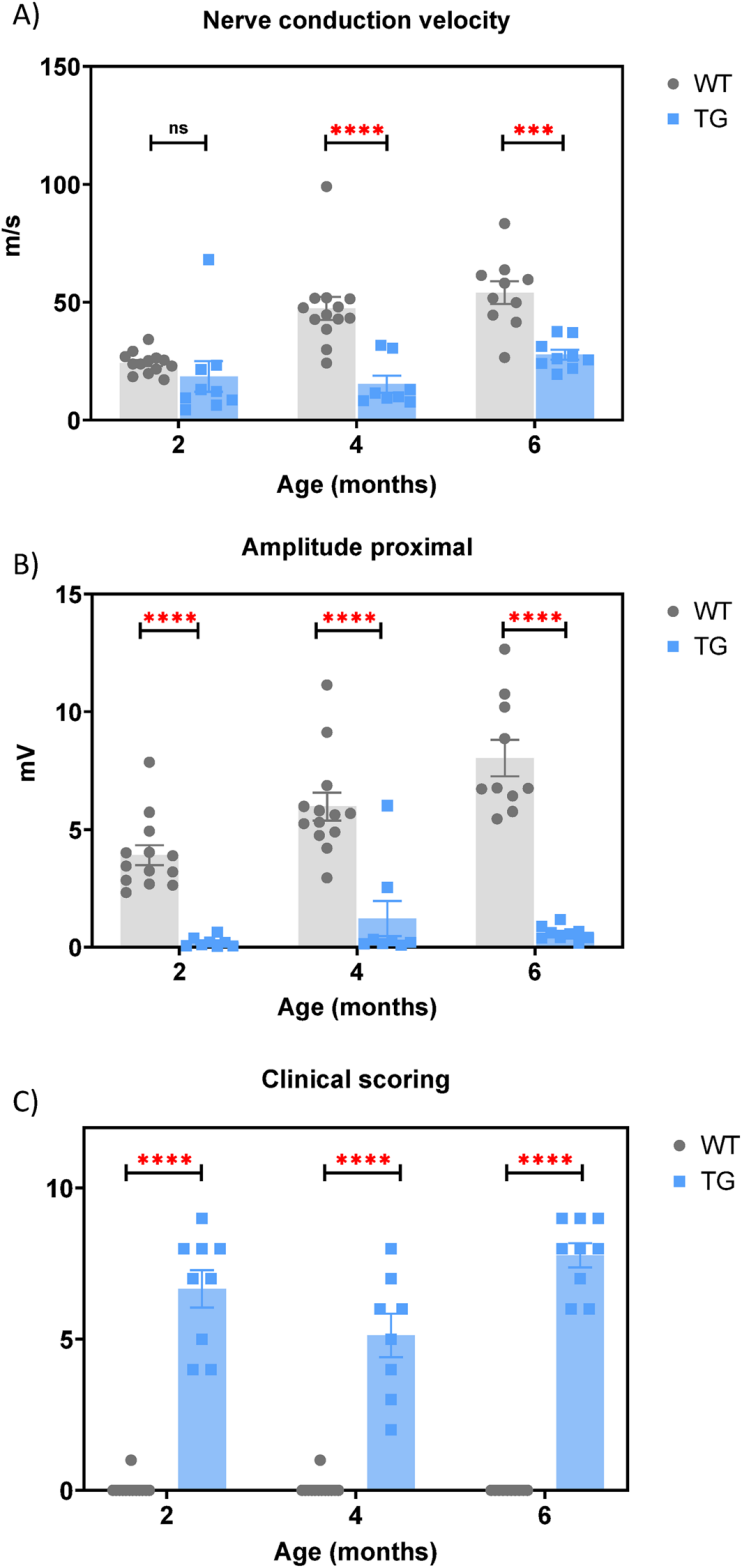
Nerve conduction analyses and clinical scoring of WT and TG rats at the age of 2, 4 and 6 months (*n* = 13, 10, 10 in WT rats and *n* = 9, 7, 9 in TG rats in the 2- and 4-month-old groups, respectively): **(A)** nerve conduction velocity; **(B)** signal amplitude of the proximal site; **(C)** clinical scoring.

Body weight monitoring, clinical scoring to confirm the mild phenotype of the model, and nerve conduction studies were performed a few days prior to sampling in 2- and 4-month groups, and longitudinally in the 6-month group. The nerve conduction velocity, signal amplitude, and clinical scoring in TG rats showed distinct expected phenotypes compared to wild-type (WT) rats (Fig. 1A–C). Specifically, TG rats showed significantly slower nerve conduction velocity (with only a trend observed at 2 months), severely reduced signal amplitude due to severe dysmyelination, and therefore markedly affected clinical phenotypes as indicated by clinical scoring. The overall profile of the nerve conduction exhibited a typical pattern of dysmyelination, as previously described by Sereda et al^25^..

### Distinct metabolic phenotypes in the sciatic nerve tissue of PMP22 TG rats

**Fig. 2:**
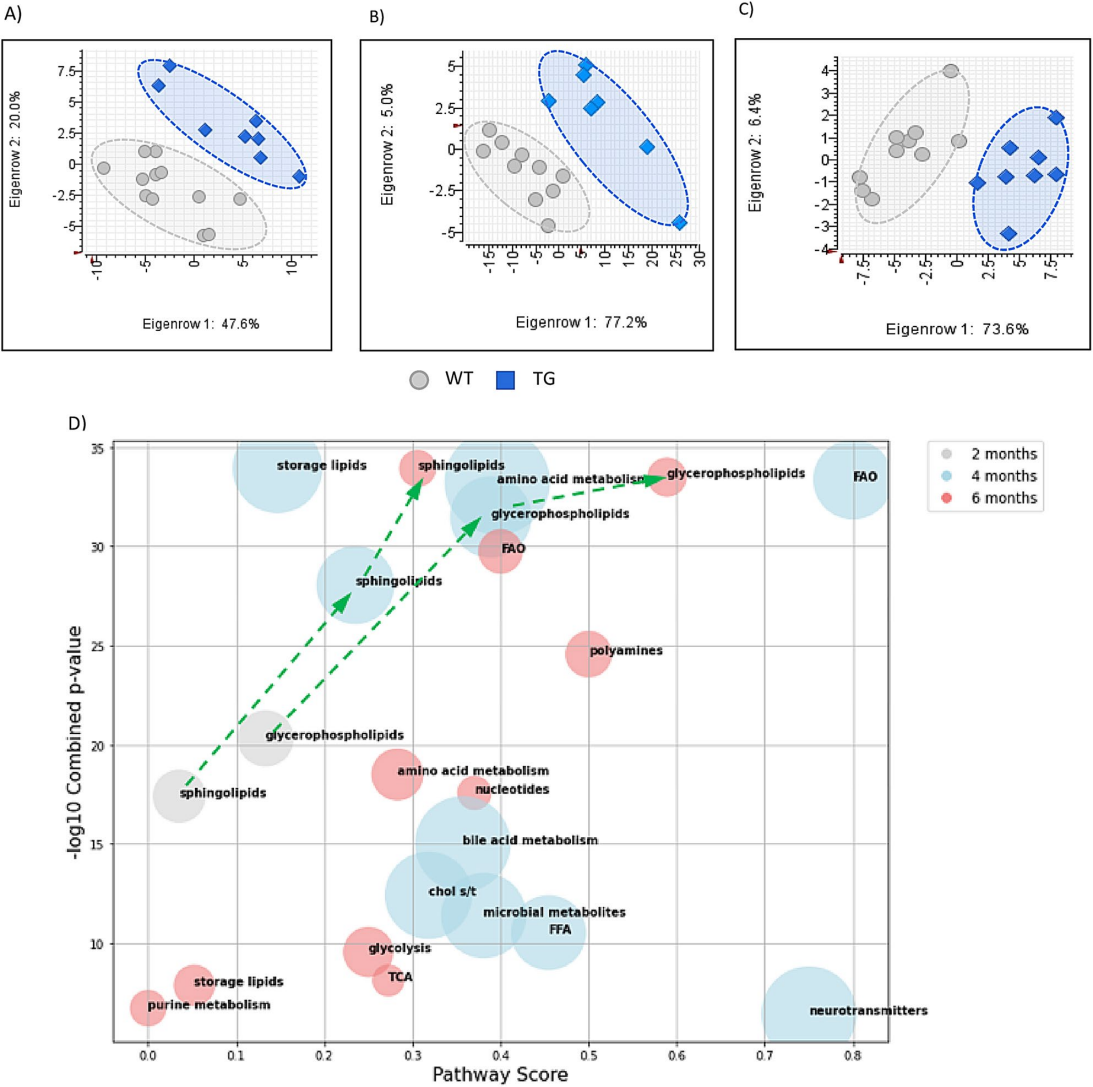
**A)–C)** Principal component analysis (PCA) of metabolic profiles from sciatic nerve tissue of female WT and TG rats at 2, 4, and 6 months of age. **D)** Bubble plot illustrating significantly altered metabolic pathways or metabolite classes in sciatic nerve tissue across different ages (2, 4, and 6 months). Pathways examined include glycolysis, tricarboxylic acid (TCA) cycle, amino acid metabolism, bile acid metabolism, cholesterol storage and transport (chol s/t), fatty acid oxidation (FAO), glycerophospholipids, sphingolipids, storage lipids, microbial metabolites, neurotransmitters, nucleotides, free fatty acids (FFA), and polyamines. Pathway scores represent the proportion of significantly altered metabolites relative to the total number of metabolites analyzed within each pathway. Combined *p*-values were calculated using Fisher’s test. Bubble size represents the average effect size, weighted by *p*-values within each pathway. Green dotted arrows indicate the directional trend of alterations in sphingolipid and glycerophospholipid levels with increasing age.

At the ages of 2, 4, and 6 months, plasma and sciatic nerve tissue were collected for targeted metabolic profiling, focusing on central metabolic pathway intermediates and lipids. Sciatic nerve tissue was chosen for the analysis because the CMT1A pathology is length-dependent, and the sciatic nerve is severely affected in this model. The metabolic profiles of sciatic nerve tissue from PMP22 TG rats were distinctly different from those of WT rats at the same age (Fig. 2A–C). The metabolic pathways contributing most significantly to this differentiation are illustrated in the bubble plot (Fig. 2D). At 2 months of age, TG rats exhibited significant changes exclusively in sphingolipids and glycerophospholipids. These alterations persisted and remained significant at both 4 and 6 months. With increasing age, more lipid species were found to undergo significant changes, as indicated with the increasing pathway scores. The pathway score increased as more significantly changed metabolites within the pathway were detected. This progression is marked by the dotted green lines with arrows.

In addition to lipids, starting at 4 months and continuing through 6 months, significant changes were also observed in diverse metabolic pathways, including energy metabolism (glycolysis, the TCA cycle, and the pentose phosphate pathway), as well as amino acids, polyamines, nucleotides, storage lipids, cholesterol storage/transport, and microbial metabolites. These findings suggest a broader and more complex dysregulation of metabolic pathways as the animals mature. A complete list of significantly changed metabolites is provided in Supplementary Table S2.

### Alteration in lipid metabolism across different ages

**Fig. 3:**
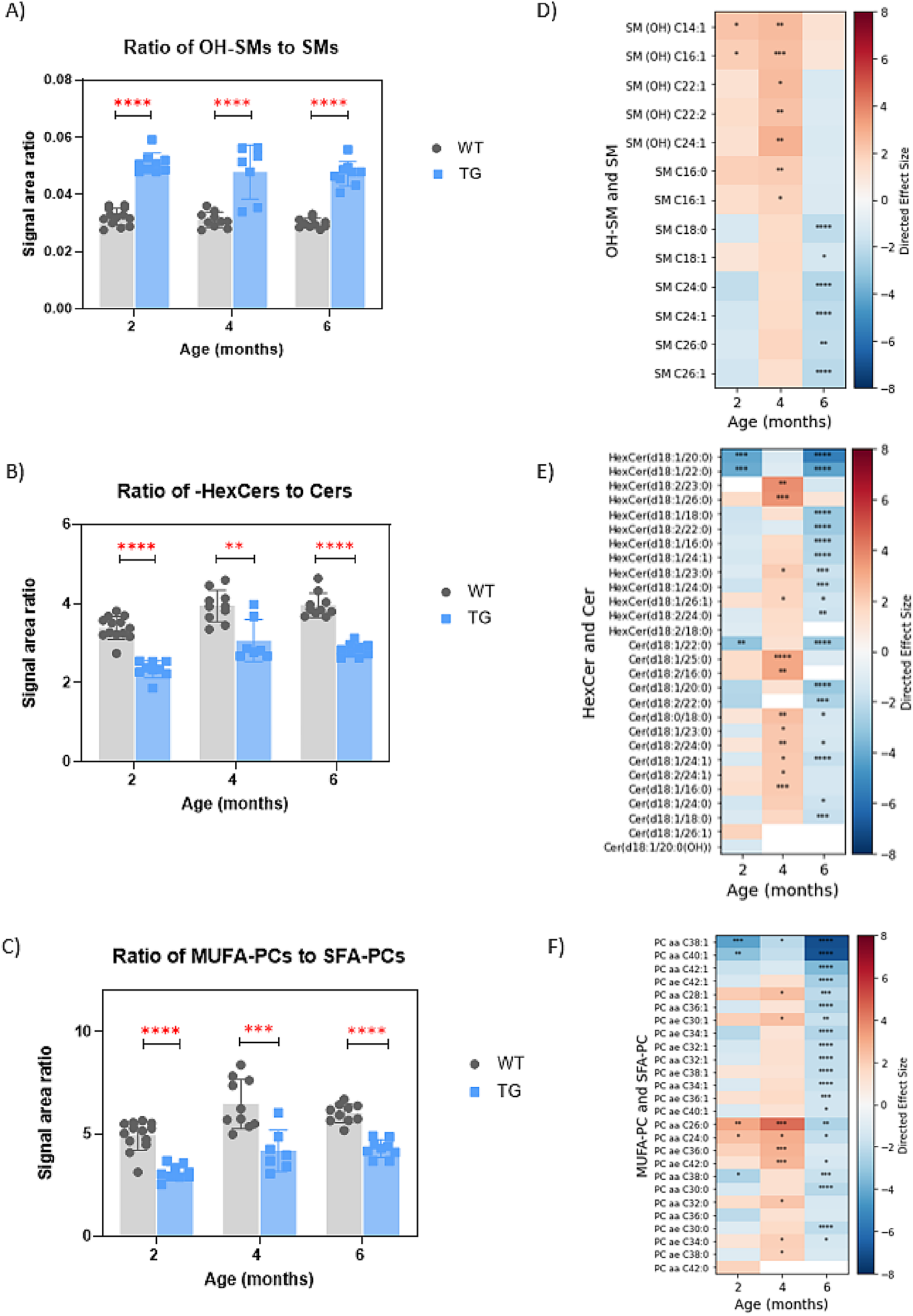
Lipid signatures in sciatic nerve tissues from female WT and TG rats. **(A)** Ratio of total hydroxysphingomyelins to total non-hydroxysphingomyelin species (OH-SMs/SMs); **(B)** Ratio of total hexosylceramide to ceramide species (HexCers/Cers); **(C)** Ratio of total monounsaturated fatty acid-containing phosphatidylcholine species to saturated fatty acid-containing phosphatidylcholine species (MUFA-PCs/SFA-PCs); **D)–F)** Heatmaps of directed effect size showing individual metabolite changes in TG group compared to the WT group for OH-SMs, SMs, HexCers, Cers, SFA-PCs, and MUFA-PCs at 2, 4, and 6 months of age. Metabolites with significant changes are marked with asterisks (*).

Among the various metabolic pathway alterations observed, the most consistent changes across all ages occurred in sphingolipids and glycerophospholipids. In TG rats, these changes include an increased ratio of hydroxylated sphingomyelins compared to non-hydroxylated sphingomyelins (SM-OHs/SMs), a decreased ratio of hexosylceramides to ceramides (HexCers/Cers), and a decreased ratio of total phosphatidylcholines (PCs) with monounsaturated fatty acid (MUFA) to those with saturated fatty acid (SFA) (MUFA-PCs/SFA-PCs) (Fig. 3 A and B, and 3 C, respectively).

In this study, a total of 15 sphingomyelins with acyl chains containing even-numbered 16 to 26 carbons were analyzed (Supplementary Table S1), including five hydroxylated sphingomyelins. Significant increases in the SM-OHs/SMs ratio were largely driven by elevated levels of SM-OH species, particularly SM (OH) C14:1 and SM (OH) C16:1 at both 2 and 4 months of age. However, by 6 months, most sphingomyelin species were decreased in TG compared to WT, except for SM (OH) C14:1 and SM (OH) C16:1 (Fig. 3D). Despite these changes in individual species, the SM-OHs/SMs ratio remained significantly higher in the TG group than in the WT group across all three ages (Fig. 3 A).

**Fig. 4:**
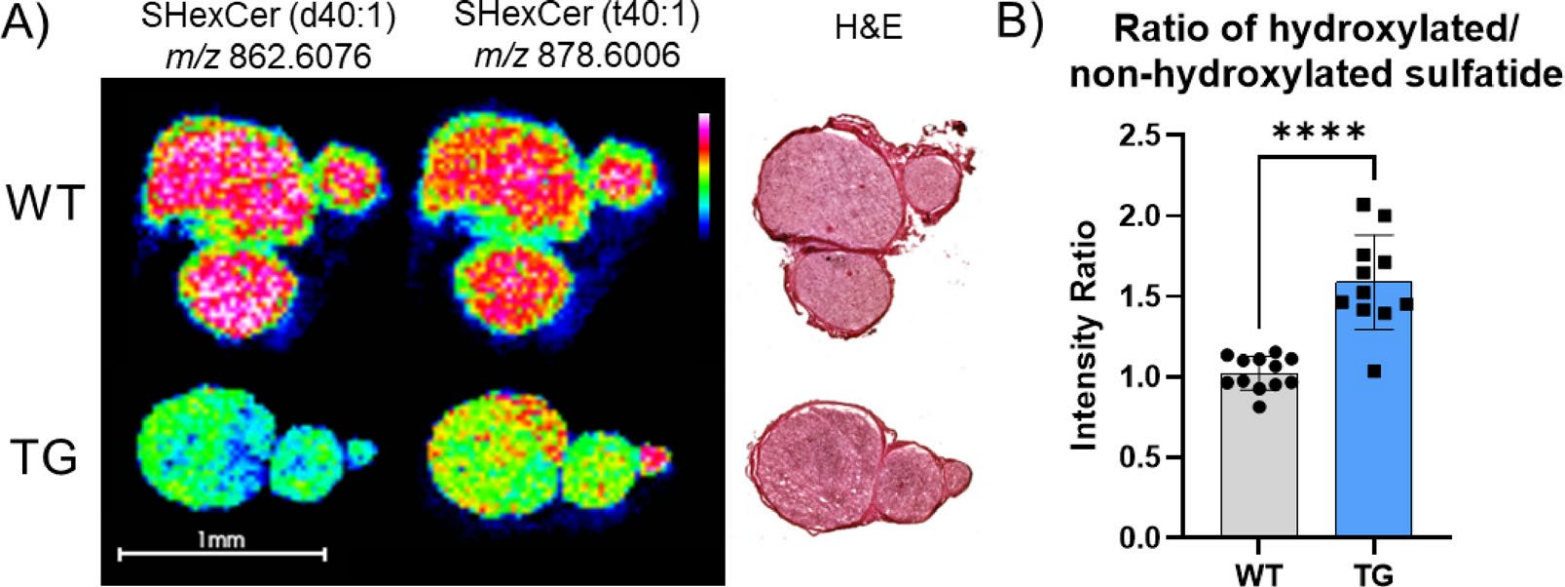
MALDI imaging mass spectrometry (IMS) analysis of a sphingolipid subclass, sulfatides, in the sciatic nerve of WT and TG rats at four months of age. **(A)** IMS images of m/z 862.6076, corresponding to a sulfatide species (sulfated hexosylceramides (d40:1)), and m/z 878.6006, a hydroxylated sulfatide species (sulfated hexosylceramides (t40:1)), respectively; **(B)** Ratio of the hydroxylated sulfatide to the non-hydroxylated sulfatide across all samples.

In addition to SMs, increased hydroxylation was observed in another sphingolipid subtype, sulfatides, by MALDI imaging MS analysis. While individual sulfatide species were found to be either increased (Fig. 4 A) or decreased (Supplementary Figure S2A) in TG sciatic nerve tissue, the ratio of a given hydroxylated sulfatide to the corresponding non-hydroxylated form was consistently increased in TG rat nerve tissues (Fig. 4B and Supplementary Figure S2B).

In the case of hexosylceramides, 36 ceramide and 19 hexosylceramide species were analyzed. The analytical method used in this study does not differentiate between galactosylceramide and glucosylceramide. This means that detected hexosylceramides represent a combined total of both subtypes. Unless otherwise specified, we use the broader term “hexosylceramide” throughout this study. At 2 months of age, ceramide levels were altered, with some species being increased and others decreased in TG rats (Fig. 3E). By 4 months, most ceramide species were significantly increased in the TG group, yet the HexCers/Cers ratio remained lower in the TG group compared to the WT group. At 6 months, most ceramide species were decreased in the TG rats, while the HexCers/Cers ratio consistently remained lower across all ages (Fig. 3B).

A total of 71 phosphatidylcholine species were detected in sciatic nerve tissue, with carbon numbers ranging from 22 to 44 and up to six double bonds. The significantly decreased ratio of MUFA-PCs to SFA-PCs was primarily driven by a significantly decreased level of long- to very-long-chain MUFA-PCs. Similar to sphingolipids, PC species showed age-dependent changes: at 2 months, some PC species were increased while others were decreased in TG rats. At 4 months, most PC species were increased in the TG rats, except for a few long- to very-long-chain MUFA-PCs. By 6 months, most PC species were decreased in the TG rats (Fig. 3 F). Despite age-dependent changes in individual PC species, the MUFA-PCs to SFA-PCs ratio consistently remained lower in TG rats compared to WT across all ages (Fig. 3 C). This trend was further supported by MALDI imaging data (Figure S3). No significant change in polyunsaturated fatty acid (PUFA)-PCs to SFA-PCs ratio was detected (data not shown).

### Plasma metabolic profile in TG rats compared to WT

**Fig. 5:**
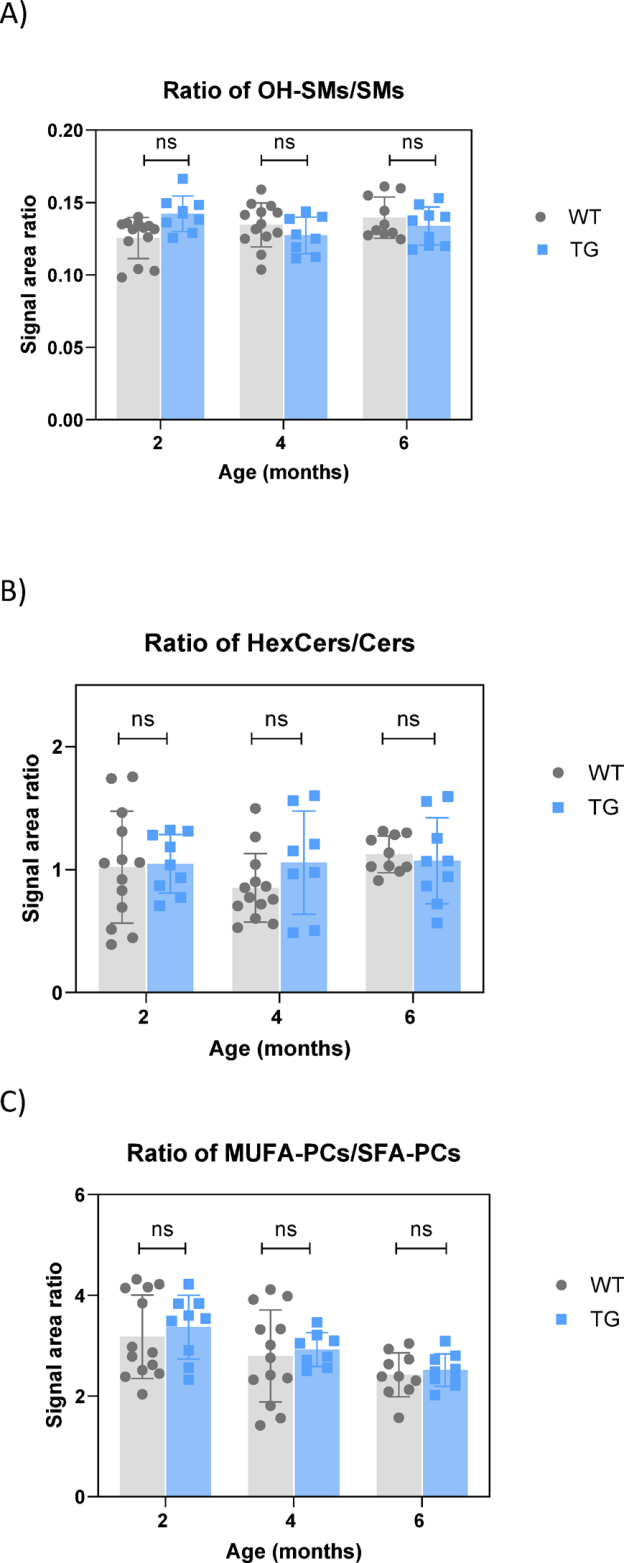
Lipid signatures in plasma from female rat. **(A)** OH-SMs/SMs; **(B)** HexCers/Cers; **(C)** MUFA-PCs/SFA-PCs.

Using the same targeted metabolomic panels applied to sciatic nerve tissue, over 500 metabolites were detected in rat plasma, including more than 300 lipid species. Plasma metabolic profiles showed no significant differences between WT and TG groups across all ages, as demonstrated by the PCA plots (Supplementary Figure S4A–C and Table S3). The lipid signatures identified in the sciatic nerve tissue were not significantly altered in plasma samples (Fig. 5A–C).

## Discussion

It has been well demonstrated that CMT1A is a dysmyelinating form rather than a demyelinating one, as historically described^18,26^. Dysmyelination refers to developmental abnormalities in myelin formation, whereas demyelination characterizes the loss of pre-existing myelin^7,18,26^. It is also important to note that dysmyelination causes abnormal nerve function, secondary inflammation, and may ultimately lead to secondary demyelination over the course of disease^7,26^.

The aim of this study was to identify the metabolic signatures in the sciatic nerve tissue over time using a PMP22 transgenic rat model, in order to detect the earliest possible metabolic changes. These signatures could serve as pharmacodynamic readouts for investigating early pharmacological interventions. Sereda et al., who initially developed this transgenic rat model, demonstrated that CMT1A is caused by transcriptional overexpression of PMP22. This overexpression results in hypomyelination, leading to phenotypes such as impaired electrophysiology, gait abnormalities, and muscle weakness^25^. They observed features of peripheral neuropathy, including reduced nerve conduction velocities (an electrophysiological hallmark), which are highly similar to those clinical findings in patients with CMT1A.

Our metabolomics data revealed a distinct metabolic profile in sciatic nerve tissue from PMP22 TG rats compared to WT controls across all ages. Early, persistent, and progressive phospholipid alterations were observed. As the animals aged, metabolic changes became more complex, with the most significant changes observed at 4 months of age (Fig. 2D). The observations in phospholipids are consistent with those of Visigalli et al., who reported similar alteration of individual SP and GP lipid species in sciatic nerve tissue of PMP22 TG rats at 2 months and in human CMT1A^27^. The observed changes in phospholipids may be linked to impaired cell membrane integrity and signaling. This aligns with the mechanism reviewed by Hertzog et al., which describes that impaired myelin lipid biosynthesis leads to imbalanced lipid/protein stoichiometry, ultimately causing hypomyelination and dysmyelination^18^.

In addition to substantial alterations in SPs and GPLs across all ages, significant changes were also observed in other lipid classes at older ages, including cholesterol esters, bile acids, intermediates of fatty acid oxidation (acylcarnitines), free fatty acids, and storage lipids, including triacylglycerols (TAG) and diacylglycerols (DAG). Since myelin in PNS is formed by Schwann cells, these results align with those of Prior et al., who reported that PMP22 downregulates genes involved in lipid and cholesterol metabolism. This dysregulation disrupts lipid homeostasis, storage, and the composition of plasma membrane lipids in Schwann cells derived from iPSCs of CMT1A patients^16^. Fledrich et al. similarly identified a significant reduction of lipids in myelin-enriched sciatic nerve tissue from PMP22 TG rats. These lipid changes were linked to the CMT1A phenotype and could be rescued by phospholipid therapy^22^.

While most lipid alterations varied with age, three specific readouts (OH-SMs/SMs, HexCers/Cers, and MUFA-PCs/SFA-PCs) remained age-independent. To our knowledge, these signatures have not been reported previously in the context of CMT1A and will be discussed in detail below.

### Increased ratio of hydroxylated sphingolipids to non-hydroxylated sphingolipids

Sphingomyelin and cerebroside (glycosphingolipids) are the major components in myelin^17,19,28^. Hydroxylation occurs at both long-chain base (sphingoid base) at the C3 or C4 position, as well as at the N-linked acyl chain at the C2, C3, or w-C position^29,30^. Among these, 2-hydroxylation in the N-linked acyl residue of sphingomyelin is the most common and an important constituent of the myelin sheath^19,30^. Hydroxylation of sphingolipids has many biological implications, such as modification of plasma membrane properties and maintaining the stability of the myelin sheath in neural tissue^31–33^. Changes in the ratio of hydroxylation to non-hydroxylated SM in nerve tissue can have significant consequences on membrane stability, myelin integrity, and ultimately the nerve function. Ekholm et al. showed that 2-hydroxlation of SM affects intermolecular packing and enhances the thermostability of membrane domain^33^. Similarly, Dan et al. reported that increased hydroxylation can improve the stability and compactness of myelin, which is essential for efficient nerve impulse transmission. However, excessive hydroxylation can disrupt the normal balance of lipids in myelin and potentially affect nerve signal transduction^34^. Fatty acid 2-hydroxylase (FA2H) catalyzes the 2-hydroxylation of sphingolipids^35^, and mutations in the FA2H gene have been implicated in a variety of human diseases, particularly in several neurodegenerative disorders^30,36,37^.

In the current study, we found that the levels of individual lipid species of both hydroxylated sphingolipids and their corresponding non-hydroxylated forms were age-dependent. However, the hydroxylation ratio (OH-SMs/SMs and hydroxylated/non-hydroxylated sulfatides) consistently increased in TG animals across all examined ages (Fig. 3 A for SMs and Fig. 4B for sulfatides). Early research by Visigalli et al. reported a significant decrease in SM species containing 16:0, 18:0, 24:0, and 24:1 fatty acid residues in sciatic nerve tissue of TG rats with CMT1A at 2 months of age^27^. In the current work however, we observed a significant decrease in individual SM species in TG rats only at older ages (Fig. 3B). Age-dependent alterations in individual lipid species were also reported in a recent study by Capodivento et al., which described lipid alterations in purified peripheral CMT1A myelin extracted from sciatic nerve tissue to be already present at postnatal day (P) 5 and prominent between P10 and P20^38^. This suggests that the lipid profile from the myelin-enriched fraction provides a much more sensitive readout for dysmyelination compared to the analyses of the whole sciatic nerve tissue, where lipid alteration may be diluted by lipids from other cell types and tissues, including blood vessels.

Notably, the significant alteration of the OH-SMs/SMs ratio, observed at 2 months of age in the sciatic nerve tissue, suggests that sphingolipid hydroxylation could be a sensitive and consistent readout for dysmyelination and related disease phenotypes. This observation is further supported by our MALDI imaging MS data, which demonstrate a significant increase in the hydroxylated/non-hydroxylated sulfatide ratio in the nerve tissue of TG rats. Future studies should focus on monitoring the ratio between hydroxylated sphingolipid versus non-hydroxylated sphingolipid species in myelin enriched tissue fraction at the earliest possible timepoint to identify the onset of lipid alterations and understand their correlation with myelin formation.

### Decreased ratio of hexosylceramides to ceramides

Hexosylated ceramides, also known as glycosylceramides, are synthesized through the addition of a sugar molecule to ceramide. The most common hexosylceramides are galactosylceramide and glucosylceramide. Galactosylceramide is a major component of the myelin sheath, comprising approximately 10–17% of myelin lipids^17,19,28^.

An imbalanced hexosylceramide-to-ceramide ratio may have significant implications for nerve tissues, particularly in terms of the structure, stability, and function of the myelin sheath^18,39^. In CMT1A myelin, both ceramide and hexosylceramide were reported to show significant reductions^17,27^. Similar to the findings for SMs, significant decreases in individual ceramide and hexosylceramide levels were observed only in the sciatic nerve tissue from TG rats at 6 months of age.

Furthermore, deficiency in the enzyme galactosylceramidase (GALC), which is critical for glycosylceramide catabolism, has been linked to dysmyelination and severe neurodegeneration disorders^39^. More broadly, disruptions in sphingolipid metabolism, i.e., the balance in HexCers/Cers ratio, may contribute to CMT1A pathophysiology by impairing myelin stability and nerve function.

Despite the important role in maintaining myelin structure and functionality, glycosphingolipids are dispensable for initial myelin formation. For example, studies of UDP-galactose: ceramide galactosyltransferase (CGalT)-deficient mice revealed that glycosphingolipid reduction in oligodendrocytes could be partially compensated by the increased synthesis of hydroxylated sphingomyelin^40^. However, whether Schwann cells exhibit a similar compensatory mechanism has not been reported. In this study, we observed a decreased HexCers/Cers ratio coinciding with an increased OH-SM/SM ratio. Whether this is the compensatory mechanism requires further investigation.

### Decreased ratio of MUFA-PCs to SFA-PCs

Phosphatidylcholine constitutes approximately 10% of total lipids and is an important structural component of the PNS myelin sheath (reviewed by Poitelon et al^17^.). Myelin lipids are predominantly highly saturated. Both saturation and long acyl chain length play crucial roles in maintaining the stability of the myelin sheath. A reduced ratio of MUFA-PCs to SFA-PCs may impact plasma membrane fluidity and alter other cellular functions in nerve tissues undergoing dysmyelination^28^. A study by Naffaa et al. shed light on the role of MUFAs during myelin development and degradation by reporting an increased proportion of MUFA in phospholipid subclasses, including PCs, during the myelination process in the CNS. However, it remains unclear whether the proportion of MUFAs decreases during demyelination^42^. Zhou et al. demonstrated that rapid demyelination in mice due to oligodendrocytes depletion of Quaking (Qki) protein, a key regulator of myelin lipid homeostasis, is accompanied by a significant reduction in MUFA-containing myelin lipids^43^. Mutations in the qki gene have been shown to lead to dysmyelination^44,45^. Furthermore, Bogie et al. highlighted the impact of MUFAs on demyelinating disorders mediated by stearoyl-CoA desaturase-1 (SCD1) in macrophages and glial cells^46^. While these findings suggest potential links between MUFAs and myelin formation processes, further mechanistic investigations are needed to reveal the underlying biology, potential consequences, and possible root causes of the alteration in fatty acid composition during dysmyelination in CMT1A.

### Metabolic signatures in sciatic nerve tissue

The overexpression of PMP22 leads to misfolding and accumulation of the protein in Schwann cells, resulting in endoplasmic reticulum (ER) retention and activation of the unfolded protein response (UPR)^47,48^. Under this condition, Schwann cells may undergo metabolic reprogramming or rewiring by favoring proper protein folding and degradation to maintain myelination. As a consequence, biosynthetic pathways for fatty acids, cholesterol, and phospholipids that are required for myelin sheath formation, may become disrupted. In addition, mitochondrial function and cellular energy homeostasis can be further impaired. This energetic stress, including energy deficiency, may negatively impact axonal transport and communication, which have high energy demands^49^.

Significant alterations in amino acid metabolism were observed at 4 months and 6 months. These changes suggest possible associations with key cellular processes including: (1) Myelin lipid biosynthesis with changes in serine for sphingolipid synthesis and glycine for supporting phosphatidylcholine synthesis; (2) Oxidative stress response, reflected by elevated arginine methylation and sulfur-containing amino acids, including methionine, methionine sulfoxide, cystine, homocysteine, and taurine synthesis; and (3) Repair and regeneration, e.g., increased biogenic amines derived from ornithine (Supplementary Table S2).

The myelin lipid biosynthesis pathways can be disrupted by defects in amino acid metabolism, leading to destabilization of myelin and impaired remyelination^17,50,51^. Restoring amino acid supply can rescue myelin lipid synthesis^52^. For example, L-serine is a central precursor for sphingolipid biosynthesis. It is the required substrate for serine palmitoyltransferase (SPT), which initiates the first rate-limiting step of sphingolipid biosynthesis^53^. L-serine treatment in the CMT1A rat model improved CMT1A myelin internode formation^27^. In the current study, reduced serine levels at 6 months may indicate a decline in sphingolipid biosynthesis (Supplementary Table S2).

Significant alterations in energy metabolism were observed, including mitochondrial fatty acid β-oxidation (elevated medium-chain acylcarnitines), TCA cycle intermediates (elevated citric acid and cis-aconitic acid), storage lipids (elevated TAGs/DAGs and cholesterol esters). Alterations in storage lipids and free fatty acids have also been reported in the CMT1A myelin fractions during postnatal development from day 5 to day 180^38^. Elevated levels of glycolysis intermediates, including glucose and lactate, were also observed at 4 months but declined at 6 months, along with TCA cycle intermediates, such as citric acid and itaconic acid. Metabolic changes at 6 months were generally less pronounced, with fewer significantly changed metabolites compared to those at 4 months, suggesting a decline in the compensatory effect due to the prolonged disruption of multiple cellular processes.

These findings support the notion that Schwann cells may undergo metabolic reprogramming to mitigate the effects of PMP22 overexpression, resulting in dysregulated lipid synthesis and metabolism, oxidative stress, and energy deficiency. Many of the metabolic changes identified in this study are reported for the first time. Understanding their potential impact and biological implications in Schwann cells and myelination requires future research, particularly focusing on the metabolic pathways involving myelin-specific lipid synthesis, oxidative stress response, redox capacity for repair and regeneration, as well as energy metabolism and homeostasis.

### Plasma metabolic profile

Visigalli et al. reported that alterations in glycerophospholipid and sphingolipid metabolism, detected in the myelin-enriched fraction and sciatic nerve tissue of CMT1A transgenic rats, could also be identified in the serum from both rats and CMT1A patients. Their finding highlights the potential of phospholipids as translational biomarkers^27^. However, the data provided by Visigalli et al. suggested that changes at the pathway level, rather than at the level of individual phospholipid species observed in nerve tissue, were reflected in cerebrospinal fluid (CSF) or in serum.

Visigalli et al. found 67 features with putative identities in rat serum related to significant alterations in glycerophospholipid metabolism using an unbiased lipidomics analysis. These features included lipid species from lipid classes such as phosphatidic acid (PA), phosphatidylethanolamine (PE), phosphatidylserine (PS), and phosphatidylinositol (PI). These lipid species were among the primary drivers of the lipid signatures identified in rat serum^27^. However, these lipid species could not be monitored using current targeted metabolomics panels, which may explain why no significant alterations were detected in the rat plasma in the current study.

This highlights the need for future studies to expand targeted metabolomics panels to include a broader range of lipid classes, such as PA, PE, PS, and PI, by applying more advanced targeted lipidomics analysis^54^. Furthermore, potential differences introduced by using plasma versus serum should be carefully investigated in future experimental designs^55,56^.

Beyond technological aspects, the likelihood of detecting local tissue metabolite changes in circulation depends on numerous factors. While Visigalli et al. demonstrated that the lipid profile in circulation is consistent with that observed in enriched myelin fractions at the lipid class and pathway level, our study specifically aimed to identify individual lipid species that are significantly altered in both nerve tissue and plasma. However, individual lipid species in circulation originate from various sources, potentially diluting the effects of myelin-specific lipid alterations at the level of individual species.

## Conclusion

In conclusion, our study reveals that sciatic nerve tissue in CMT1A rat model undergoes significant changes, not only in lipid metabolism but also in other metabolic pathways, including energy and amino acid metabolism. These changes exhibit a dynamic nature at the metabolite level that may represent primary or secondary effects resulting from the overexpression of PMP22, or could arise from a combination of biological processes related to growth, aging, and disease progression.

Notably, we report, for the first time, consistent alterations in the hydroxylation and glycosylation of sphingolipids, as well as saturation/desaturation of glycerophospholipids in the sciatic nerves of CMT1A rats across different ages. These striking lipid signatures emerge at 2 months of age and persist throughout maturation. Both LC-MS/MS and IMS analyses conducted on sciatic nerve tissue confirmed these findings.

While these lipid signatures might reflect the intrinsic regulatory role of PMP22 in lipid metabolism, changes in other metabolic pathways may arise from adaptive and compensatory responses depending on the stages of physical development. Further investigations are required to elucidate the underlying mechanisms driving these changes and to explore their functional consequences in the context of CMT1A pathophysiology.

## Materials and methods

### Animals

The PMP22 transgenic rat model^25^ was licensed from Prof. Dr. Klaus-Armin Nave at the Max Planck Institute for Multidisciplinary Sciences. Breeding and genotyping were performed at Charles River Laboratories Germany GmbH (Sulzfeld, Germany). The wild-type rats were littermates. Both male and female animals were included. The rats were acclimatized to the facility for at least 7 days, housed in groups of two to three animals at 25 °C with a 12:12-hour light-dark cycle, and fed a standard laboratory diet containing 18.2% protein and 3.0% fat with an energy content of 15.8 MJ/kg (KLIBA NAFAG 3890, GRANOVIT AG, Kaiseraugst, Switzerland). Food and water were provided *ad libitum*. The experiments described here were performed on license BS-2885 approved by the Cantonal Veterinary Office according to the regulations effective in the Canton of Basel-City, Switzerland. This study is reported in accordance with ARRIVE guidelines. All methods were performed in accordance with the relevant guidelines and regulations in Switzerland.

To characterize the PMP22 transgenic rat model, the following clinical scoring scales were applied, and a sum of clinical scores was calculated: unsteady gait (0–2), steppage gait (0–2), outward hindfoot positioning (0–2), walking on whole feet (0–2), jumpiness (0–1), inability to right up on stretched hindlimb (0–1), swing difficulties (0–1), hindlimb weakness (0–2), tremors (0–2), piloerection (0–1).

To measure motor nerve conduction velocity and amplitude (baseline to peak), rats were anesthetized with isoflurane and the hind limb fur was shaved with an electric razor. The sciatic and tibial nerves were then stimulated with monopolar needle electrodes inserted into the sciatic notch and the hock using an ADInstruments’ electrical stimulator setup. Data were acquired with PowerLab system, and motor nerve conduction velocity and amplitude were analyzed using LabChartPro (ADInstruments GmbH, Spechbach, Germany).

Clinical scoring, body weight measurement, and nerve conduction studies were performed longitudinally in the 6-month-old groups. In the 2- and 4-month-old groups, clinical scoring and body weight measurement were performed a few days before necropsy. The number of rats per group was as follows: *n* = 13, 10, 10 in WT group and *n* = 9, 7, 9 in TG group with ages of 2-, 4-, and 6-months, respectively. For the MALDI-IMS analysis, a separate cohort of WT rats(*n* = 12) and TG rats (*n* = 12) at 4-months of age was used, distinct from those used for targeted metabolomics analysis. Values for nerve conduction parameters and clinical scores are expressed as mean ± SEM. Statistical analysis was carried out to compare WT and TG at the age of 2, 4, and 6 months of age, using Šídák’s multiple comparisons test following two-way ANOVA. Differences were considered statistically significant at *p* < 0.05; ***: *p* < 0.001, ****: *p* < 0.0001, ns: not significant. Statistical analyses were performed using GraphPad Prism (GraphPad Software, La Jolla, CA, USA).

### Collection of plasma and sciatic nerve samples

At the end of the experiment, rats were euthanized with CO_2_ (> 85 vol%) followed by blood collection from the abdominal vein. EDTA-plasma samples were prepared from the collected blood. Sciatic nerve samples were collected from the euthanized rats, immediately frozen in liquid nitrogen, and stored at −80 °C until targeted metabolomics analysis.

### Targeted metabolic profiling

More than 600 metabolites were analyzed in sciatic nerve tissue and plasma samples using targeted LC-MS/MS methods, specifically the CCM (central carbon metabolism) panel and the MxP^®^ Quant 500 panel (Biocrates life sciences AG, Innsbruck, Austria). These metabolites originate from various metabolic pathways or classes, including glycolysis, the tricarboxylic acid (TCA) cycle, the pentose phosphate pathway (PPP), nucleotide metabolism, nucleotide sugars, purine metabolism, amino acid metabolism, polyamines, free fatty acids (FFA), fatty acid oxidation (FAO), steroid hormones, cholesterol storage and transport, bile acids, glycerophospholipids, sphingolipids, storage lipids, co-factors, and microbial-derived metabolites. A complete list of metabolites, along with their corresponding pathways or metabolite classes (collectively referred to as “pathways” hereafter for simplicity) is provided in Supplementary Table S1 and summarized in Figure S1. In addition to the metabolite levels determined by LC-MS/MS, over 200 “metabolic indicators” were calculated. These indicators include sums of metabolites within a single pathway and ratios between different metabolites. The sums and ratios were calculated using the MetaboINDICATOR™ software tool. For example, the ratio SM-OHs/SMs = SUM(SM-OH)/SUM(SM), where SUM(SM-OH) = SM (OH) C14:1 + SM (OH) C16:1 + SM (OH) C22:1 + SM (OH) C22:2 + SM (OH) C24:1, SUM(SM) = SM C16:0 + SM C16:1 + SM C18:0 + SM C18:1 + SM C20:2 + SM C22:3 + SM C24:0 + SM C24:1 + SM C26:0 + SM C26:1. More details are provided in Table S1. Both summed values and ratios were statistically compared between age-matched TG and WT groups.

### IC-MS/MS for the CCM panel analysis

An ion exchange chromatography (IC) system coupled to tandem mass spectrometer (MS/MS) was used to analyze polar metabolites involved in CCM pathways including glycolysis, TCA cycle, PPP, nucleotides, nucleotide sugars, short chain fatty acids, microbial metabolites, and other organic and inorganic acids. Metabolite standards and isotopically labelled standards were purchased from Sigma Aldrich, Switzerland. Milli-Q water with 18.2 MΩ·cm was generated by a PureLab Ultra water purification system (Elga-Veolia, Plainfield, IL, USA). Methanol (ULC/MS grade) was purchased from Biosolve Chemicals (Biosolve Chimie, France). The complete list of metabolites and internal standards is provided in the supplementary material.

For the CCM panel analysis of plasma samples, 10 µL of plasma was extracted with 90 µL of 90% methanol containing all internal standards at a concentration of 30.55 nM. Samples were vortexed for 10 min at 1000 rpm followed by centrifugation at 16,000 x g for 20 min at 4 °C. The supernatants were transferred to new tubes, evaporated under nitrogen at 40 °C, reconstituted in 200 µL of water and vortexed for 10 min. The reconstituted extracts were filtered using UltraFree™ MC filter tubes (Merck Millipore, 0.1 μm) by centrifugation at 12,000 x g for 90 min. Finally, filtered extracts were transferred into polypropylene autosampler injection vials (Infochroma 8002-SC-PP3µ) with 100 µL inserts for IC-MS/MS analyses.

For sciatic nerve tissue, each pre-weighed nerve sample was transferred to a 7 mL Precellys^®^ tube containing twenty zirconium beads (2.8 mm diameter). Four milliliters of 90% methanol containing internal standards at a concentration of 13.75 nM were added to each tube. Nerve tissue was homogenized with a Precellys^®^ Evolution homogenizer (Bertin Technologies, Montigny-le-Bretonneux, France), cooled on dry ice, with the following settings: 27 cycles of 20 s at 6800 rpm with a 10-second rest interval between cycles. The homogenate was transferred to 5 mL Eppendorf tubes and centrifuged at 16,000 x g for 20 min. The supernatant was evaporated under nitrogen at 40 °C. The residue was reconstituted in 100 µL of water, shaken for 10 min at 1500 rpm, and centrifuged at 16,000 x g for 15 min at 4 °C before filtration using Ultrafree™ MC filters (Millipore, Merck & Cie, Darmstadt, Germany) at 12,000 x g for 60 min. The filtered extracts were transferred to HPLC vials for IC-MS/MS analysis.

CCM panel analyses were conducted using a Dionex™ ICS-5000 + reagent-free HPIC™ high-pressure anion exchange system (Thermo Scientific, Olten, Switzerland) coupled to a Sciex QTrap^TM^6500+ mass spectrometer (AB Sciex LLC, Framingham, MA, USA), equipped with an electrospray ionization (ESI) source. Chromatographic separation was achieved using an IonPac AS-18 column (0.4 × 150 mm, 4 μm particle size) and an IonPac AG-18 guard column (0.4 × 35 mm, 4 μm particle) (Thermo Scientific, Reinach, Switzerland) at a flow rate of 17 µL/min. The column temperature was maintained at 35 °C. Elution was performed using a potassium hydroxide gradient generated by an eluent generator with the following program: 0 min at 10 mM, 1 min at 33 mM, 13.5 min at 36 mM, 23.7 min at 117 mM, 28.1 min at 117 mM, 28.2 min at 10 mM, and 32 min at 10 mM. Following column separation, an ion suppressor was operated in an external water circulating mode with a regeneration flow rate of 22 µL/min and a current of 25 mA. A make-up flow of 9 µL/min methanol was added to the IC flow prior to the ESI source to enhance ionization efficiency. Milli-Q water (18.2 MΩ·cm) was used for the eluent generation and regeneration of ion suppressor regeneration. The MS was operated in negative ion mode with multiple reaction monitoring (MRM) mode for detection. Metabolite levels in arbitrary units (a.u.) were calculated by normalizing signal areas to the corresponding internal standards (IS). Chromatogram peaks were detected and integrated using MultiQuan™ software (AB Sciex LLC, Framingham, MA, USA), and resulting data were exported for statistical analysis.

### MxP^®^ Quant500 panel analysis

A total of 107 small molecules and 523 lipids were analyzed using a Sciex QTrap^TM^6500 mass-spectrometer (AB Sciex LLC, Framingham, MA, USA) coupled with LC-MS/MS operated in chromatography separation and flow injection analysis (FIA) modes. The Quant500 MxP™ kit (Biocrates Life Sciences AG, Innsbruck, Austria) was used as one of the two targeted metabolic profiling methods. Plasma and tissue extraction was according to the protocol instruction provided with the kit. LC-MS/MS peak integration and FIA data analysis were conducted using the MetIDQ™ software (Biocrates Life Sciences AG, Innsbruck, Austria). Processed data was exported from MedIDQ™ for further statistical analysis. MetaboINDICATOR™ software tool, included as part of MedIDQ™ software package, was used to calculate the metabolite sums and ratios.

*Matrix-assisted laser desorption/ionization imaging mass spectrometry (MALDI IMS) analysis*.

Sciatic nerves were collected and embedded in carboxymethyl cellulose prior to snap-freezing in liquid nitrogen. The sciatic nerve tissue was sectioned at 10 μm thickness using a CM3050 S cryostat (Leica Biosystems). Sections were thaw mounted onto indium tin oxide (ITO) coated microscope slides for MALDI IMS. A 1,5-Diaminonaphthalene (DAN) matrix was prepared at 5 mg/mL in 90% acetonitrile and applied using a TM-Sprayer instrument (HTX Technologies) with the following settings: matrix flow rate of 0.10 mL/min, stage speed of 1300 mm/min, 10 passes, 75 °C, and a 3 mm track spacing. After matrix coating, the sample plate was rehydrated in a Petri dish containing 1 mL of 70% methanol for 2 min at room temperature. MALDI imaging experiments were performed using a 7 Tesla SolariX MALDI Fourier transform ion cyclotron resonance (FTICR) mass spectrometer (Bruker Daltonics, Bremen Germany). Spectra were collected in both positive ion and negative ion mode over a range of *m/z* 150–3000. Image resolution was set to 25 μm. SCiLS Lab (Bruker Daltonics, Bremen Germany) was used for data analysis and image visualization.

### Statistical analysis

Statistical analyses and data visualization were conducted using Analyst™ in Genedata Expressionist^®^ (version 16.0, Genedata AG, Basel, Switzerland), GraphPad Prism™ (version 10.1.2, GraphPad Software, LLC, USA), and Python 3.9 (Anaconda 3). To compare TG and WT groups across different ages, two-group unpaired t-tests were performed. Data included internal standard normalized values of each metabolite, as well as values derived from sum and ratio calculations. Prior to statistical testing, data were log10-transformed to approximate normal distributions. Unless otherwise stated, Student’s *t*-tests were applied throughout the study. Effect size was calculated using group means, and the Benjamini-Hochberg critical value (BH-Q value) was determined for each *p*-value. Robust changes were considered significant if they met both criteria: *p*-value < 0.05 and a false discovery rate (FDR or BH-Q value) < 0.05. Statistical significance was indicated in figures using asterisk markers as follows: * *p* < 0.05, ** *p* < 0.01, *** *p* < 0.001, and **** *p* < 0.0001, with “ns” denoting no significance. A detailed list of effect sizes, *p*-values, and BH-Q-values are listed in Supplementary Table S2. Principal component analysis (PCA) was conducted using both the full set of instrumentally acquired (> 600) and calculated ratios and sums (> 300), as well as a subset of metabolites selected based on *p* -values < 0.05. Metabolites detected in fewer than 50% of the samples in either group (TG or WT) at any age were excluded from the statistical analysis. To assess pathway impact and aggregate the significance of metabolites within each pathway, pathway scores were calculated by counting significant metabolites relative to the total number of metabolites analyzed within that pathway or metabolite class. Aggregation of *p*-values across pathways was performed using Fisher’s combined probability test, with a BH-Q value cut-off of 0.05.

## Supplementary Information

Below is the link to the electronic supplementary material.

Supplementary Material 1

Supplementary Material 2

Supplementary Material 3

Supplementary Material 4

## Data Availability

In addition to the supplementary information, further data supporting the findings of this work, such as processed targeted metabolomics and imaging mass spectrometry data, nerve conduction parameters and clinical scores, are available from the corresponding author upon reasonable request.

## References

[1] Saporta, M. A. & Shy, M. E. Inherited peripheral neuropathies. *Neurol. Clin.* **31**, 597–619. 10.1016/j.ncl.2013.01.009 (2013). 23642725 10.1016/j.ncl.2013.01.009 PMC3646296

[2] DiVincenzo, C. et al. The allelic spectrum of Charcot-Marie-Tooth disease in over 17,000 individuals with neuropathy. *Mol. Genet. Genomic Med.* **2**, 522–529. 10.1002/mgg3.106 (2014). 25614874 10.1002/mgg3.106 PMC4303222

[3] Morena, J., Gupta, A. & Hoyle, J. C. Charcot-Marie-Tooth: from molecules to therapy. *Int. J. Mol. Sci.* **20**10.3390/ijms20143419 (2019). 10.3390/ijms20143419 PMC6679156 31336816

[4] Eichinger, K. et al. Accelerate clinical trials in Charcot-Marie-Tooth disease (ACT-CMT): A protocol to address clinical trial readiness in CMT1A. *Front. Neurol.* **13**, 930435. 10.3389/fneur.2022.930435 (2022). 35832173 10.3389/fneur.2022.930435 PMC9271780

[5] Bolino, A. & D’Antonio, M. Recent advances in the treatment of Charcot-Marie-Tooth neuropathies. *J. Peripher Nerv. Syst.* **28**, 134–149. 10.1111/jns.12539 (2023). 36855793 10.1111/jns.12539

[6] Okamoto, Y. & Takashima, H. The current state of Charcot-Marie-Tooth disease treatment. *Genes (Basel)*. 14. 10.3390/genes14071391 (2023). 10.3390/genes14071391 PMC10379063 37510296

[7] Li, J. Caveats in the established Understanding of CMT1A. *Ann. Clin. Transl Neurol.* **4**, 601–607. 10.1002/acn3.432 (2017). 28812050 10.1002/acn3.432 PMC5553227

[8] Patel, P. I. et al. The gene for the peripheral Myelin Protein-Pmp-22 is a candidate for Charcot-Marie-Tooth disease Type-1a. *Nat. Genet.* **1**, 159–165. 10.1038/ng0692-159 (1992). 1303228 10.1038/ng0692-159

[9] Timmerman, V. et al. The peripheral Myelin protein gene Pmp-22 is contained within the Charcot-Marie-Tooth disease Type-1a duplication. *Nat. Genet.* **1**, 171–175. 10.1038/ng0692-171 (1992). 1303230 10.1038/ng0692-171

[10] Valentijn, L. J. et al. The peripheral Myelin gene Pmp-22/Gas-3 is duplicated in Charcot-Marie-Tooth disease Type-1a. *Nat. Genet.* **1**, 166–170. 10.1038/ng0692-166 (1992). 1303229 10.1038/ng0692-166

[11] Murphy, S. M. et al. Charcot-Marie-Tooth disease: frequency of genetic subtypes and guidelines for genetic testing. *J. Neurol. Neurosurg. Psychiatry*. **83**, 706–710. 10.1136/jnnp-2012-302451 (2012). 22577229 10.1136/jnnp-2012-302451 PMC3736805

[12] Snipes, G. J., Welcher, S. U. & Shooter, A. A. Characterization of a novel peripheral nervous system Myelin protein (PMP22/SR13). *J Cell. Biol. Apr*. **117** (1), 225–238. 10.1083/jcb.117.1.225 (1992). 10.1083/jcb.117.1.225 PMC2289391 1556154

[13] Adlkofer, K. et al. Hypermyelination and demyelinating peripheral neuropathy in Pmp22-deficient mice. *Nat. Genet.* **11**, 274–280. 10.1038/ng1195-274 (1995). 7581450 10.1038/ng1195-274

[14] Jetten, A. M. & Suter, U. The peripheral Myelin protein 22 and epithelial membrane protein family. *Prog Nucleic Acid Res. Mol. Biol.* **64**, 97–129. 10.1016/s0079-6603(00)64003-5 (2000). 10697408 10.1016/s0079-6603(00)64003-5

[15] Mittendorf, K. F. et al. Peripheral myelin protein 22 alters membrane architecture. *Sci Adv* 3 (2017). https://doi.org/ARTN e170022010.1126/sciadv.1700220. 10.1126/sciadv.1700220 PMC5498104 28695207

[16] Prior, R. et al. Defective Schwann cell lipid metabolism alters plasma membrane dynamics in Charcot-Marie-Tooth disease 1A. *bioRxiv* (2023). 10.1101/2023.04.02.535224

[17] Poitelon, Y., Kopec, A. M. & Belin, S. Myelin fat facts: an overview of lipids and fatty acid metabolism. *Cells* **9**10.3390/cells9040812 (2020). 10.3390/cells9040812 PMC7226731 32230947

[18] Hertzog, N. & Jacob, C. Mechanisms and treatment strategies of demyelinating and dysmyelinating Charcot-Marie-Tooth disease. *Neural Regeneration Res.* **18**, 1931–1939. 10.4103/1673-5374.367834 (2023). 10.4103/1673-5374.367834 PMC10233759 36926710

[19] Schmitt, S., Castelvetri, L. C. & Simons, M. Metabolism and functions of lipids in Myelin. *Biochim. Biophys. Acta*. **1851**, 999–1005. 10.1016/j.bbalip.2014.12.016 (2015). 25542507 10.1016/j.bbalip.2014.12.016

[20] Bais, P. et al. Metabolite profile of a mouse model of Charcot-Marie-Tooth type 2D neuropathy: implications for disease mechanisms and interventions. *Biol. Open.* **5**, 908–920. 10.1242/bio.019273 (2016). 27288508 10.1242/bio.019273 PMC4958279

[21] Soldevilla, B. et al. Plasma metabolome and skin proteins in Charcot-Marie-Tooth 1A patients. *PLoS One*. **12**, e0178376. 10.1371/journal.pone.0178376 (2017). 28575008 10.1371/journal.pone.0178376 PMC5456076

[22] Fledrich, R. et al. Targeting Myelin lipid metabolism as a potential therapeutic strategy in a model of CMT1A neuropathy. *Nat. Commun.* **9**, 3025. 10.1038/s41467-018-05420-0 (2018). 30072689 10.1038/s41467-018-05420-0 PMC6072747

[23] Setlere, S. et al. Metabolomics insights into Charcot-Marie-Tooth disease: toward biomarker discovery. *Front. Neurol.* **16**, 1543547. 10.3389/fneur.2025.1543547 (2025). 40458460 10.3389/fneur.2025.1543547 PMC12127190

[24] Bustos, A. et al. Metabolic and functional improvements in a patient with Charcot-Marie-Tooth disease type 2 after EGCG administration: A case report. *Med. (Kaunas)*. **57**, 10.3390/medicina57020104 (2021). 10.3390/medicina57020104 PMC7912075 33498819

[25] Sereda, M. et al. A Transgenic rat model of Charcot-Marie-Tooth disease. *Neuron* **16**, 1049–1060. 10.1016/s0896-6273(00)80128-2 (1996). 8630243 10.1016/s0896-6273(00)80128-2

[26] Fridman, V. & Saporta, M. A. Mechanisms and treatments in demyelinating CMT. *Neurotherapeutics* **18**, 2236–2268. 10.1007/s13311-021-01145-z (2021). https://doi.org/34750751 10.1007/s13311-021-01145-z PMC8804145

[27] Visigalli, D. et al. Exploiting Sphingo- and glycerophospholipid impairment to select effective drugs and biomarkers for CMT1A. *Front. Neurol.* **11**, 903. 10.3389/fneur.2020.00903 (2020). 32982928 10.3389/fneur.2020.00903 PMC7477391

[28] O’Brien, J. Stability of Myeline membrane. *Science ***147***, *1099–1107*. *10.1126/science.147.3662.1099 (1965). 10.1126/science.147.3662.1099 14242030

[29] Kawana, M., Miyamoto, M., Ohno, Y. & Kihara, A. Comparative profiling and comprehensive quantification of stratum corneum ceramides in humans and mice by LC/MS/MS. *J. Lipid Res.* **61**, 884–895. 10.1194/jlr.RA120000671 (2020). 32265320 10.1194/jlr.RA120000671 PMC7269764

[30] Eckhardt, M. & Fatty Acid 2-Hydroxylase and 2-Hydroxylated sphingolipids: metabolism and function in health and diseases. *Int. J. Mol. Sci.* **24**10.3390/ijms24054908 (2023). 10.3390/ijms24054908 PMC10002949 36902339

[31] Marques, J. T., Marinho, H. S. & de Almeida, R. F. M. Sphingolipid hydroxylation in mammals, yeast and plants - An integrated view. *Prog Lipid Res.* **71**, 18–42. 10.1016/j.plipres.2018.05.001 (2018). 29746894 10.1016/j.plipres.2018.05.001

[32] Hama, H. Fatty acid 2-Hydroxylation in mammalian sphingolipid biology. *Biochim. Biophys. Acta*. **1801**, 405–414. 10.1016/j.bbalip.2009.12.004 (2010). 20026285 10.1016/j.bbalip.2009.12.004 PMC2826524

[33] Ekholm, O., Jaikishan, S., Lonnfors, M., Nyholm, T. K. & Slotte, J. P. Membrane bilayer properties of sphingomyelins with amide-linked 2- or 3-hydroxylated fatty acids. *Biochim. Biophys. Acta*. **1808**, 727–732. 10.1016/j.bbamem.2010.12.006 (2011). 21167130 10.1016/j.bbamem.2010.12.006

[34] Dan, P., Edvardson, S., Bielawski, J., Hama, H. & Saada, A. 2-Hydroxylated sphingomyelin profiles in cells from patients with mutated fatty acid 2-hydroxylase. *Lipids Health Dis.* **10**, 84. 10.1186/1476-511X-10-84 (2011). 21599921 10.1186/1476-511X-10-84 PMC3107802

[35] Alderson, N. L. et al. The human FA2H gene encodes a fatty acid 2-hydroxylase. *J. Biol. Chem.* **279**, 48562–48568. 10.1074/jbc.M406649200 (2004). 15337768 10.1074/jbc.M406649200

[36] German, A. et al. Novel homozygous FA2H variant causing the full spectrum of fatty acid Hydroxylase-Associated neurodegeneration (SPG35). *Genes (Basel)*. 15. 10.3390/genes15010014 (2023). 10.3390/genes15010014 PMC10815826 38275596

[37] Dan, P., Edvardson, S., Bielawski, J., Hama, H. & Saada A. 2-hydroxylated sphingomyelin profiles in cells from patients with mutated fatty acid 2-hydroxylase. *Lipids Health Dis. ***10**, 84. https://doi.org/10.1186/1476-511X-10-84 (2011). 10.1186/1476-511X-10-84 PMC3107802 21599921

[38] Capodivento, G. et al. Monitoring Myelin lipid composition and the structure of myelinated fibers reveals a maturation delay in CMT1A. *Int. J. Mol. Sci.* **25**10.3390/ijms252011244 (2024). 10.3390/ijms252011244 PMC11508568 39457026

[39] Reza, S., Ugorski, M. & Suchanski, J. Glucosylceramide and galactosylceramide, small glycosphingolipids with significant impact on health and disease. *Glycobiology* **31**, 1416–1434. 10.1093/glycob/cwab046 (2021). 34080016 10.1093/glycob/cwab046 PMC8684486

[40] Saadat, L. et al. Absence of oligodendroglial glucosylceramide synthesis does not result in CNS Myelin abnormalities or alter the dysmyelinating phenotype of CGT-Deficient mice. *Glia* **58**, 391–398. 10.1002/glia.20930 (2010). 19705459 10.1002/glia.20930 PMC2807477

[41] Hornemann, T. Mini review: lipids in peripheral nerve disorders. *Neurosci. Lett.* **740**, 135455. 10.1016/j.neulet.2020.135455 (2021). 33166639 10.1016/j.neulet.2020.135455

[42] Naffaa, V. et al. Shift in phospholipid and fatty acid contents accompanies brain myelination. *Biochimie* **203**, 20–31. 10.1016/j.biochi.2022.08.010 (2022). 36055603 10.1016/j.biochi.2022.08.010

[43] Zhou, X. et al. Mature Myelin maintenance requires Qki to coactivate PPARbeta-RXRalpha-mediated lipid metabolism. *J. Clin. Invest.* **130**, 2220–2236. 10.1172/JCI131800 (2020). 32202512 10.1172/JCI131800 PMC7191000

[44] Hardy, R. J. Molecular defects in the dysmyelinating mutant quaking. *J. Neurosci. Res.* **51** (19980215), 417–422. 10.1002/(SICI)1097-4547 (1998). ::AID-JNR1>3.0.CO;2-F 9514195 10.1002/(SICI)1097-4547(19980215)51:4<417::AID-JNR1>3.0.CO;2-F

[45] Zhao, L., Tian, D., Xia, M., Macklin, W. B. & Feng, Y. Rescuing QkV dysmyelination by a single isoform of the selective RNA-binding protein QKI. *J. Neurosci.* **26**, 11278–11286. 10.1523/JNEUROSCI.2677-06.2006 (2006). 17079655 10.1523/JNEUROSCI.2677-06.2006 PMC6674528

[46] Bogie, J. F. J. et al. Stearoyl-CoA desaturase-1 impairs the reparative properties of macrophages and microglia in the brain. *J Exp Med* 217 (2020). https://doi.org/ARTN e2019166010.1084/jem.20191660. 10.1084/jem.20191660 PMC7201924 32097464

[47] Hara, T. et al. Rer1 and calnexin regulate Endoplasmic reticulum retention of a peripheral Myelin protein 22 mutant that causes type 1A Charcot-Marie-Tooth disease. *Sci. Rep.* **4**, 6992. 10.1038/srep06992 (2014). 25385046 10.1038/srep06992 PMC4227013

[48] Bai, Y. et al. Treatment with IFB-088 improves neuropathy in CMT1A and CMT1B mice. *Mol. Neurobiol.* **59**, 4159–4178. 10.1007/s12035-022-02838-y (2022). 35501630 10.1007/s12035-022-02838-y PMC9167212

[49] Nave, K. A. Myelination and the trophic support of long axons. *Nat. Rev. Neurosci.* **11**, 275–283. 10.1038/nrn2797 (2010). 20216548 10.1038/nrn2797

[50] Hu, J. et al. Myeloid cell-associated aromatic amino acid metabolism facilitates CNS Myelin regeneration. *NPJ Regen Med.* **9**, 1. 10.1038/s41536-023-00345-9 (2024). 38167866 10.1038/s41536-023-00345-9 PMC10762216

[51] de Koning, T. J. Amino acid synthesis deficiencies. *J. Inherit. Metab. Dis.* **40**, 609–620. 10.1007/s10545-017-0063-1 (2017). 28653176 10.1007/s10545-017-0063-1 PMC5500668

[52] Clark, A. J. et al. An iPSC model of hereditary sensory neuropathy-1 reveals L-serine-responsive deficits in neuronal ganglioside composition and axoglial interactions. *Cell. Rep. Med.* **2**, 100345. 10.1016/j.xcrm.2021.100345 (2021). 34337561 10.1016/j.xcrm.2021.100345 PMC8324498

[53] Davis, D. L. et al. Dynamics of sphingolipids and the Serine palmitoyltransferase complex in rat oligodendrocytes during myelination. *J. Lipid Res.* **61**, 505–522. 10.1194/jlr.RA120000627 (2020). 32041816 10.1194/jlr.RA120000627 PMC7112141

[54] Anh, N. K., Thu, N. Q., Tien, N. T. N., Long, N. P. & Nguyen, H. T. Advancements in mass Spectrometry-Based targeted metabolomics and lipidomics: implications for clinical research. *Molecules* **29**, 5934 (2024). 39770023 10.3390/molecules29245934 PMC11677340

[55] Xu, R., Zhang, S., Li, J. & Zhu, J. Plasma and serum metabolic analysis of healthy adults shows characteristic profiles by subjects’ sex and age. *Metabolomics* **20**, 43. 10.1007/s11306-024-02108-z (2024). 38491253 10.1007/s11306-024-02108-z PMC10943143

[56] Vignoli, A. et al. Serum or plasma (and which plasma), that is the question. *J. Proteome Res.* **21**, 1061–1072. 10.1021/acs.jproteome.1c00935 (2022). 35271285 10.1021/acs.jproteome.1c00935 PMC8981325

