# Supplementary material for: Metabolic signatures in sciatic nerve of PMP22 transgenic rats provide insights into the pathogenesis of charcot-marie-tooth disease type 1 A
Source: Sci Rep. 2026 Jan 8;16:2036. doi: 10.1038/s41598-025-31633-7 (PMC12808651; doi:10.1038/s41598-025-31633-7)
Supplement: Supplementary file 1 — Supplementary Material 1 [file 41598_2025_31633_MOESM1_ESM.docx]

**Supplementary Figures**


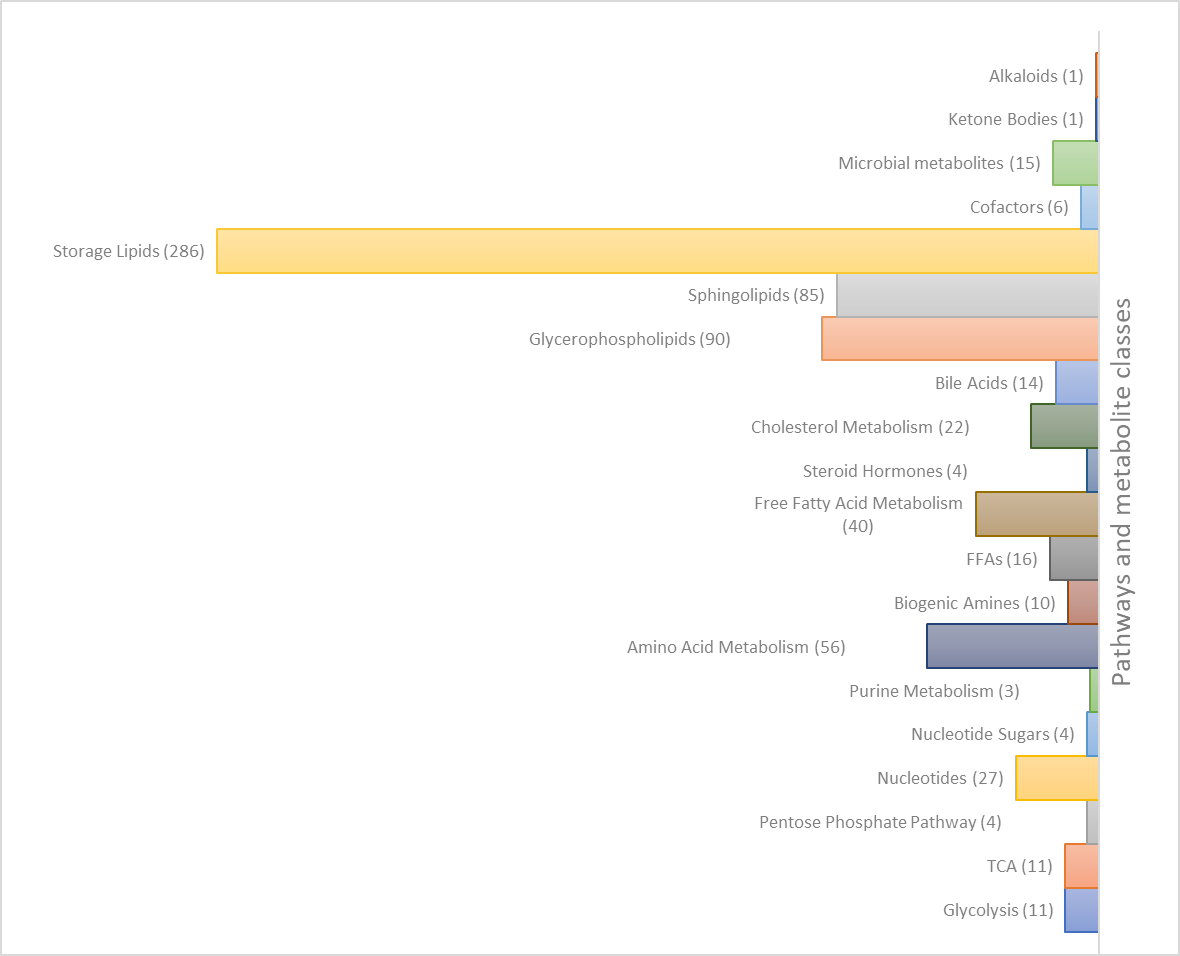


**Figure S1.** Total number of metabolites from various metabolite classes and pathways analyzed in sciatic nerve tissue and plasma using the targeted metabolomics panels, as described in the Methods section.


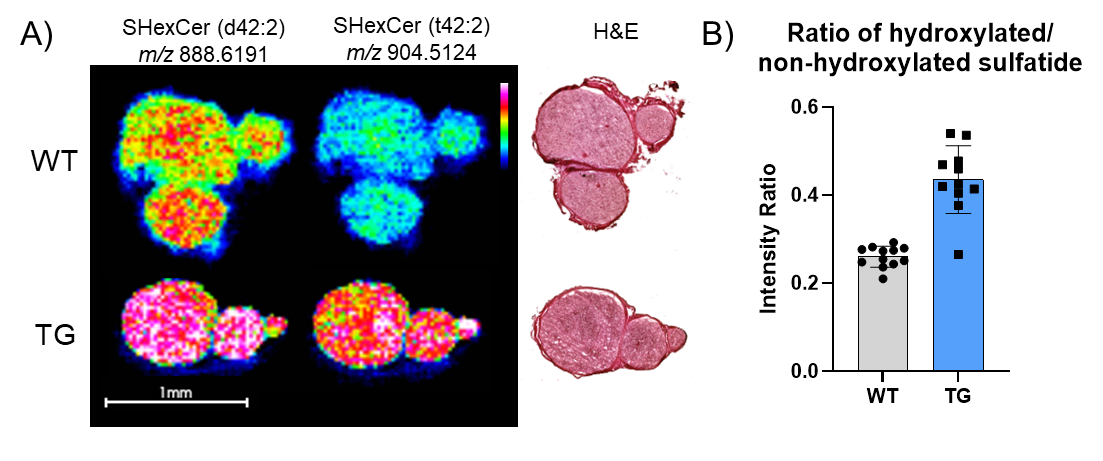


**Figure S2**. MALDI imaging mass spectrometry (IMS) analysis of sulfatides in the sciatic nerve of WT and TG rats at 4 months of age. **A)** IMS images of a sulfatide with m/z 888.6191 and its corresponding hydroxylated sulfatide with m/z 904.5124; **B)** Ratio of hydroxylated sulfatide to non-hydroxylated sulfatide for all the samples (n=12).


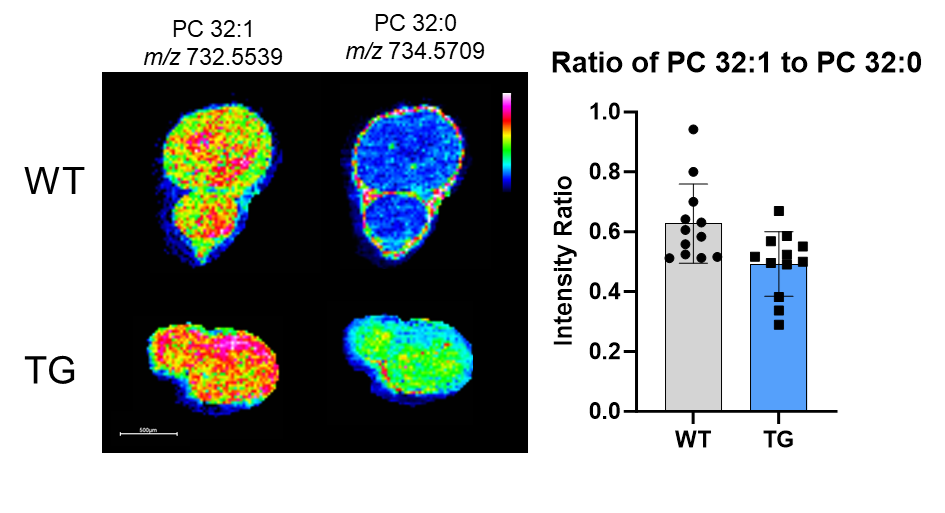


**Figure S3**. MALDI imaging mass spectrometry (IMS) analysis of phosphatidylcholines (PCs) in the sciatic nerve of WT and TG rats at 4 months of age. **A)** IMS images of m/z 732.5539, corresponding to PC 32:1, and m/z 734.5709, corresponding to PC 32:0; **B)** Ratio of monounsaturated PC 32:1 to saturated PC 32:0 across all samples (*n* = 12).

A)


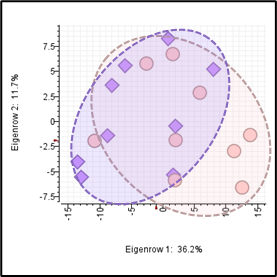

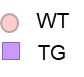


B)


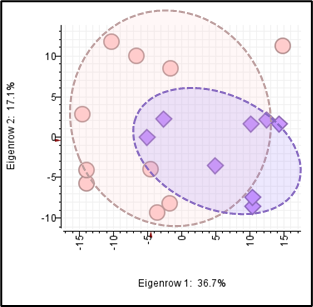


C)


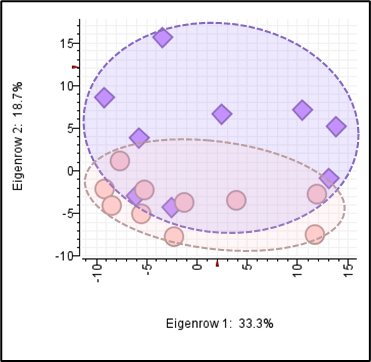


**Figure S4.** Principal component analysis of metabolic profiles obtained from rat plasma of female WT and TG rats at 2 months **A)**, 4 months **B)**, and 6 months **C)** of age.

| S5A)   |  |
| --- | --- |
| S5B)   |  |
| S5C)   |  |

**Figure S5.** Lipid signatures in sciatic nerve tissues from male WT and TG rats. **A)** Ratio of hydroxysphingomyelins to non-hydroxysphingomyelin species (OH-SMs/SMs); **B)** Ratio of hexosylceramide to ceramide species (HexCers/Cers); **C)** Ratio of phosphatidylcholine species containing monounsaturated fatty acids to those containing saturated fatty acids (MUFA-PCs/SFA-PCs).
